# *BRCA1* and *BRCA2* Gene Expression: Diurnal Variability and Influence of Shift Work

**DOI:** 10.3390/cancers11081146

**Published:** 2019-08-09

**Authors:** Massimo Bracci, Veronica Ciarapica, Maria Eléxpuru Zabaleta, Maria Fiorella Tartaglione, Silvia Pirozzi, Letizia Giuliani, Francesco Piva, Matteo Valentino, Caterina Ledda, Venerando Rapisarda, Richard G. Stevens, Lory Santarelli

**Affiliations:** 1 Occupational Medicine, Department of Clinical and Molecular Sciences, Polytechnic University of Marche, 60126 Ancona, Italy; 2 Department of Specialistic Clinical and Odontostomatological Sciences, Polytechnic University of Marche, 60126 Ancona, Italy; 3 Occupational Medicine, Department of Clinical and Experimental Medicine, University of Catania, 95124 Catania, Italy; 4 Department of Community Medicine, University of Connecticut Health Center, Farmington, CT 06032, USA

**Keywords:** *BRCA1* gene, *BRCA2* gene, DNA damage, DNA repair, breast cancer, biological clocks, shift work schedule, night shift work, chronobiology disorders, desynchronization of circadian rhythms

## Abstract

*BRCA1* and *BRCA2* genes are involved in DNA double-strand break repair and related to breast cancer. Shift work is associated with biological clock alterations and with a higher risk of breast cancer. The aim of this study was to investigate the variability of expression of *BRCA* genes through the day in healthy subjects and to measure *BRCA* expression levels in shift workers. The study was approached in two ways. First, we examined diurnal variation of *BRCA1* and *BRCA2* genes in lymphocytes of 15 volunteers over a 24-hour period. Second, we measured the expression of these genes in lymphocytes from a group of shift and daytime workers. The change in 24-hour expression levels of *BRCA1* and *BRCA2* genes was statistically significant, decreasing from the peak at midday to the lowest level at midnight. Lower levels for both genes were found in shift workers compared to daytime workers. Diurnal variability of *BRCA1* and *BRCA2* expression suggests a relation of DNA double-strand break repair system with biological clock. Lower levels of *BRCA1* and *BRCA2* found in shift workers may be one of the potential factors related to the higher risk of breast cancer.

## 1. Introduction

Increasing evidences have demonstrated diurnal variability and involvement of the biological clock in several DNA repair mechanisms [1,2,3,4,5]. In particular, in a murine model, the XPA (Xeroderma Pigmentosum A) protein, implicated in the DNA nucleotide excision repair (NER) system, showed a circadian oscillation in the brain [6]. Similarly, the HMGB1 (High mobility group box 1) protein, involved in the DNA mismatch repair (MMR), showed a diurnal oscillation in retina [7]. A diurnal modulation of OGG1 (8-oxoguanine DNA glycosylase), responsible for oxidative DNA damage repair and involved in the BER (Basic Excision Repair) system, was found in human lymphocytes by our research group [8].

Breast Cancer 1 (*BRCA1*) located on chromosome 17 and Breast Cancer 2 (*BRCA2*) located on chromosome 13 are tumor-suppressor genes involved in the DNA double-strand breaks (DBS) repair mechanisms [9]. The DBS repair systems are mainly represented by homologous recombination (HR) and by the junction of non-homologous end-joining (NHEJ) mechanisms [10,11]. BRCA1 directly participates in the HR [12,13]. Experimental evidences support a role for BRCA1 in the NHEJ repair system [14,15,16]. In addition, several studies suggest an interaction of BRCA1 with enzymes capable of modifying chromatin and DNA structure [17,18,19,20]. BRCA1 takes part in the composition of the BRCA1-Associated DNA Surveillance Complex (BASC), consisting of molecules involved in DNA repair and cell cycle control at checkpoint level [19]. In spite of the multifunctional activities of BRCA1, the primary function of BRCA2 is the repair of DSB through HR [21,22,23]. Specifically, BRCA2 mediates the recruitment of the RAD51 recombinase to DSB, which is essential for HR [24].

Mutations of the *BRCA1* and *BRCA2* genes in the germline are one of the predisposing causes for the onset of hereditary breast and ovarian cancers, as well as, prostate and pancreatic tumors [25]. In subjects carrying a germline mutation in the *BRCA1* gene, the risk of developing breast cancer is around 70–80% and for the *BRCA2* gene it is 50–60% [22].

Breast cancer is the most commonly occurring cancer in women and has the highest mortality rate among female cancers worldwide [26]. Comparing the global incidence rate of breast cancer in economically advanced regions with that in the less developed countries, data show that the former (74 per 100,000 inhabitants) is more than twice that of the latter (31 per 100,000 inhabitants) [27]. This suggests that some lifestyle factors of industrialized societies may constitute risk factors.

Accumulating evidence suggests that disruption of circadian rhythms can alter breast biology and may promote cancer [28,29]. The suppression of melatonin secretion and the alteration of the control exercised by the circadian biological clock on some pathways connected to carcinogenesis (cell cycle, DNA repair system and apoptosis) are some of the proposed mechanisms [30,31,32,33]. The increased risk of cancer could be a consequence of one or, more likely, the effect of the simultaneous action of several mechanisms [34].

In night-shift workers, periodic inversion of the sleep–wake cycle and exposure to artificial light during nighttime can result in disruption of the biological clock that in turn may be responsible for cancer [35,36,37,38]. Epidemiological studies conducted in recent years have shown an increase in the incidence and prevalence of neoplasms, particularly of breast cancer, among shift workers, suggesting a possible association between shift work and the onset of cancer [39,40,41,42,43,44]. The International Agency for Research on Cancer (IARC) re-evaluated "night shift work" as "probably carcinogenic to humans" (group 2A) [45]. In addition, persistent night-shift work that causes circadian disruption is classified as "a human carcinogen" in the draft of Report on Carcinogens Monograph on Night Shift Work and Light at Night [46].

The aim of this study was to test the variability of expression of *BRCA* genes through the day and evaluate if shift workers have altered levels of *BRCA* expression. The study was approached in two ways. First, we examined diurnal variation of *BRCA1* and *BRCA2* gene expression in lymphocytes of 15 healthy volunteers over a 24-hour period. Second, we measured the expression of *BRCA1* and *BRCA2* genes in lymphocytes from a group of shift and daytime workers by taking blood samples from all workers in the morning after a day off.

## 2. Results

### 2.1. Research in Healthy Volunteers

#### 2.1.1. Characterization of the Biological Clock

**Figure 1:**
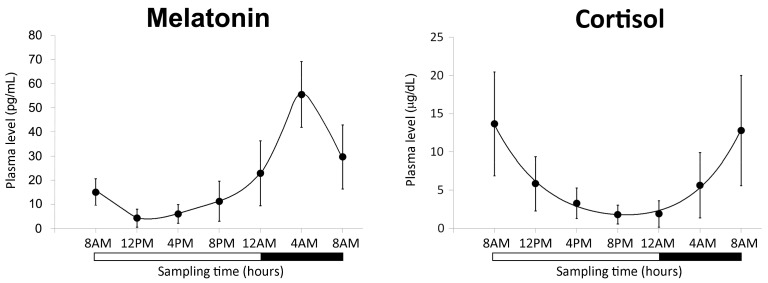
Profile of melatonin and cortisol levels (mean ± SD) in the plasma of 15 volunteers. The black region of the bar indicates the rest period at night.

The characterization of the biological clock in the group of 15 healthy subjects was performed measuring diurnal melatonin and cortisol in plasma and clock gene expression variation in lymphocytes. The 24-hour mean concentration (±SD) of plasma melatonin was 20.8 ± 17.5 pg/mL decreasing from 55.5 ± 37.9 pg/mL at 4:00 A.M. (peak) down to 5.3 ± 7.3 pg/mL at 12:00 P.M. (trough). The 24-hour mean concentration (±SD) of plasma cortisol was 6.2 ± 5.0 μg/dL, the highest value was 13.7 ± 6.8 μg/dL at 8:00 A.M., while the lowest value was 1.7 ± 1.6 μg/dL at 12:00 A.M. (Figure 1). A statistically significant variation in the 24 h was validated for melatonin and cortisol levels with ANOVA repeated measures and Cosinor analysis (*p* < 0.05).

**Figure 2:**
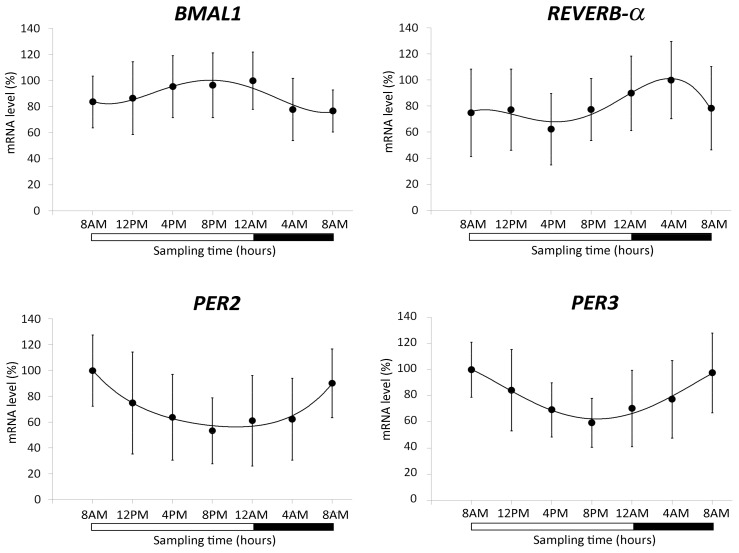
Expression profiles of clock genes (*BMAL1*, *REVERB-α*, *PER2*, and *PER3*) in lymphocytes of 15 volunteers. The mRNA levels (mean ± SD) are expressed as relative % values compared with the acrophase set at 100%. The black region of the bar indicates the rest period at night.

The characterization of the clock gene expression in the lymphocytes of the 15 volunteers was carried out investigating the expression levels of *BMAL1*, *PER2*, *PER3*, and *REVERB-α* at 4-hour intervals over a 24-hour period. All the circadian clock genes analyzed showed significant variation in the 24-hours (ANOVA repeated measures and Cosinor analysis, *p* < 0.05). Specifically, *BMAL1* showed a significant oscillation between the maximum value in the evening and the minimum value in the early hours of the morning. The *REVERB-α* gene reached the minimum value in the afternoon–evening hours and progressively increased to the maximum value in the early morning hours. The genes of the *PER* family (*PER2* and *PER3*) had an expression profile characterized by a progressive decrease from the maximum value in the morning to the minimum value in the evening (Figure 2).

#### 2.1.2. Expression Profile of *BRCA1* and *BRCA2* Genes

**Figure 3:**
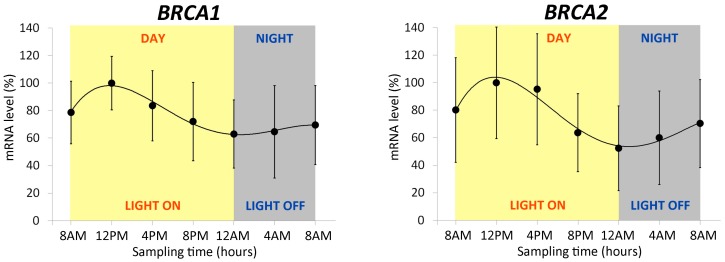
Expression profiles of *BRCA1* and *BRCA2* in lymphocytes of 15 volunteers. The mRNA levels (mean ± SD) are expressed as relative % values compared with the acrophase set at 100%.

The expression profile of *BRCA1* and *BRCA2* genes in lymphocytes was assessed by RT-qPCR at the same times of melatonin, cortisol and clock genes over a 24-hour period (Figure 3). We found a statistically significant variation in their expression from the peak at 12:00 P.M. to the lowest point at 12:00 A.M. (ANOVA repeated measures, post hoc LSD, *p* < 0.05). Cosinor analysis confirmed the ANOVA results and evidenced a period of 26.9 h and 25.3 h, and an acrophase at −171° and −184° for the oscillation of *BRCA1* and *BRCA2* expression, respectively.

### 2.2. Research in Shift and Daytime Workers

Samples were taken at 9:00 A.M., after a day off, from shift workers (*n* = 44) and daytime workers (*n* = 45) and analyzed for *BRCA1* and *BRCA2* gene expression. The number of nights worked per month by shift workers was 5.6 ± 1.3 (Table 1). A higher chronotype score, indicative of morningness preference, was found in daytime workers (*p* = 0.005), while shift workers had a higher social jet lag (*p* = 0.006). There were no significant differences shown in age, job seniority, number of smokers, BMI, and physical activity between the groups. The light exposure was investigated by a questionnaire. Non-significant differences were found between shift and daytime workers for exposure to sunlight and for the use of video display devices after dinner. A high home light intensity was more reported by daytime workers (*p* = 0.006).

**Figure 4:**
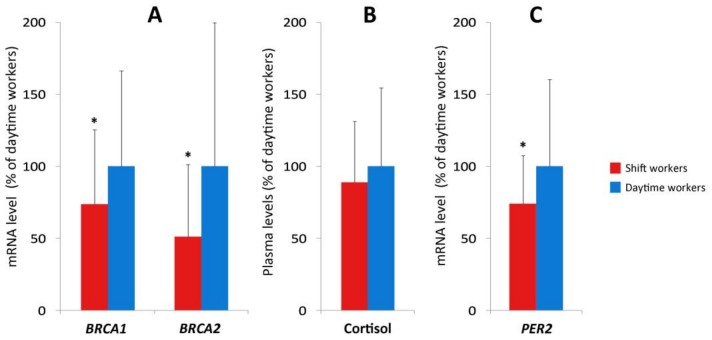
*BRCA1* and *BRCA2* values (**A**), cortisol (**B**), and *PER2* gene expression levels (**C**) in shift and daytime workers. Results (mean ± SD) are expressed as % of values of daytime workers. * = *p* < 0.05, Student’s *t*-Test for independent samples. Blood sampled in the morning after a day off from work.

Workers were investigated for cortisol and *PER2* expression as representative parameters of biological clock (Figure 4B,C). We found both parameters lower in shift workers, however, only *PER2* levels were significantly different (Student’s *t*-Test for independent samples, *p* < 0.05). Notably, we found significant lower expression levels for *BRCA1* and *BRCA2* in shift workers compared to daytime workers. The results were 1218 ± 954 vs. 1841 ± 1363 (2^−ΔCT^×10^3^) for *BRCA1* and 204 ± 203 vs. 389 ± 399 (2^−ΔCT^×10^3^) for *BRCA2* (Student’s *t*-Test for independent samples, *p* < 0.05; Figure 4A). 

We examined the correlation among these variables performing a Pearson’s analysis. As a result, we found a significant positive correlation between *BRCA1* and *BRCA2* gene expression. Both genes positively correlated with the *PER2* gene expression. In addition, we found an inverse association between *BRCA* gene expression and the number of nights worked per month (Table 2).

The multivariate analysis confirmed an effect of shift work on *BRCA1* and *BRCA2* expression (β = −0.298 *p* = 0.012 and β = −0.296 *p* = 0.012, respectively) and showed an inverse correlation of *BRCA1* and *BRCA2* levels with wake-up time on the blood sampling day (β = −0.257 *p* = 0.039; β = −0.270 *p* = 0.028, respectively) (Table 3). The effect of shift work was confirmed also on *PER2* expression (β = −0.262 *p* = 0.031).

## 3. Discussion 

Throughout evolution, the light/dark cycle determined the timing and schedule of human activity. The light phase has been associated with activity and food intake, while the dark phase is associated with rest. As a result, exposures to genotoxic agents are not constant through the day but are greater during daytime and lower during nighttime. The hypothesis that DNA repair processes may be adapted to the time of the day (i.e., greater efficiency during greater risk) has been confirmed for several DNA repair systems [4,5,8]. Among all genes involved in DNA repair pathways, *BRCA1* and *BRCA2* genes are characterized by a particular association with breast cancer [47]. Since breast cancer was also related with circadian disruption caused by shift work [39,40,41,42,43,44], we tested the hypotheses of (1) a diurnal variability of *BRCA* gene expression and (2) the influence of shift work on *BRCA* gene expression.

The expression of *BRCA1* and *BRCA2* genes was first evaluated in lymphocytes taken from healthy volunteers over a 24-hour period. Plasma melatonin and cortisol levels and selected clock gene expression (*BMAL1*, *REVERB-α*, *PER2*, and *PER3*) were analyzed to define the diurnal rhythm of the participants. We found a significant variation of plasma melatonin and cortisol levels with a typical rhythm characterized by the highest values of melatonin in the night and by a cortisol peak in the early morning [8,48,49,50,51]. Clock gene expression rhythm was the same reported in other studies [8,48,49,51]. The physiologic diurnal rhythm of the volunteers, in line with the evidence in the literature, assured that they are reasonably representative of the population.

Interestingly, *BRCA* gene expression levels showed a significant diurnal variability. Both *BRCA* genes studied had their highest levels of expression at 12:00 P.M., and the lowest at 12:00 A.M. This finding includes *BRCA1* and *BRCA2* among genes involved in DNA repair whose expression depends on the time of day [3,8]. Moreover, the diurnal variability of *BRCA1* and *BRCA2* genes suggests a direct or indirect relationship with the endogenous biological clock (e.g., *BRCA* genes could be “clock-controlled genes”). To examine this hypothesis, we analyzed the promoter region of *BRCA1* and *BRCA2* genes. Considering the amino acid sequence similarity of the basic helix-loop-helix (bHLH) domain of CLOCK and BMAL proteins between humans and mice, we searched CLOCK/BMAL1 binding motifs according to the literature [52,53,54,55] in the first 2000 bp upstream transcription start site (TSS) of *BRCA1* and *BRCA2* genes. We find one and two binding sites in *BRCA1* and *BRCA2* promoters, respectively: 1232 bp upstream TSS of *BRCA1* (gctagCACGTTgtcac), 20 bp upstream TSS of *BRCA2* (ggcgtCACGTGgccag), and 1599 bp upstream TSS of *BRCA2* (catgcCACGGGttctc). These evidences further support that *BRCA* genes could be clock-controlled genes.

In the second part of the study, the influence of shift work on *BRCA1* and *BRCA2* gene expression was investigated. Since shift work can cause perturbation of the circadian rhythm, we supposed that shift workers may have altered *BRCA1* and *BRCA2* expression levels compared to daytime workers.

Shift and daytime workers under study had similar age, tobacco habits and BMI, while chronotype and social jet lag differed between the two groups. A higher MEQ score, indicative of morningness preference, was found in daytime workers according to other studies [56,57]. Similarly, a higher social jet lag is expected in shift workers [58,59]. Regarding light exposure, no difference was found in the duration of sunlight exposure or the use of video display devices after dinner between the two groups. There was a significant difference found in the intensity of the home light. Considering the complexity to define light exposure (duration, wavelength spectrum, intensity), our results should be further studied using a more detailed questionnaire or devices able to record objective measures.

Several studies have reported that shift workers may have alterations in the parameters that characterize their biological clock [35,60,61,62]. In this study, shift workers showed a decrease in the expression of the *PER2* clock gene compared to daytime workers. This confirms an alteration of the biological clock previously observed in follicular cells of shift workers [60]. Likewise, we observed a decreased expression of both *BRCA* genes in shift workers. Since this evidence derives from just one data collection made at 9:00 A.M., we cannot discriminate whether it was related to a phase misalignment and/or a reduced phase amplitude in shift workers. Both groups where sampled after a day off, therefore we can exclude acute effects related to sleep deprivation, but a residual phase shift may be possible. Considering the reduced amplitude observed for the *PER2* gene expression in shift workers after a day off [60] and the correlation that we found between *BRCA* and *PER2* gene expression, we speculate that a reduced amplitude of *BRCA1* and *BRCA2* gene expression in shift workers is probable. Since *BRCA* genes are related to DSB repair and heterozygous mutation of *BRCA* genes results in an increased probability of breast cancer [63,64,65], the low levels of *BRCA* gene expression found in the morning in shift workers may contribute to the understanding of pathways that associate shift work and circadian clock disruption with breast cancer. Interestingly, the number of nights worked per month inversely correlated with *BRCA1* and *BRCA2* gene expression levels suggesting that there are some factors related to the night shift (e.g., light exposure at night, sleep deprivation, etc.) that particularly influence *BRCA* gene expression values.

A negative correlation between the awakening time on day of the blood sampling and *BRCA* gene expression values was found by multivariate analysis. Therefore, the earlier the workers woke up, the higher the *BRCA* gene expression levels were. This was expected because, as observed in the volunteers, the expression value of *BRCA* genes tended to increase during the morning. Taking into account the fact that the workers woke up about an hour and a half before the volunteers, the values obtained at 9:00 A.M. in the workers are probably closer to the peak than those of the volunteers at the same time. No correlation was shown between *BRCA* gene expression and chronotype, social jet lag or intensity of light at home. However, a possible correlation between *BRCA1* and the chronotype cannot be excluded because it was near the limit of significance. Moreover, chronotype is one of the main individual characteristics related to shift work tolerance [66,67]. An effect of shift work on *BRCA* gene expression levels was shown and confirmed at multivariate analysis, the statistical significance was similar for both *BRCA1* and *BRCA2* genes.

Data regarding melatonin in shift workers and its correlation with *BRCA* gene expression would have been interesting to study. The determination of the 6-sulfatoxymelatonin on urine of workers was originally planned, but since some workers delivered the second morning urine instead of the urine of the entire night, the test was not executed. The single blood sample obtained from shift workers constitutes a limitation of this study, as well as other studies on blood samples obtained from workers. Indeed, multiple withdrawals are possible to test in a small sample of volunteers, but cannot be collected from many workers.

A specific recommendation is for *BRCA* mutation carriers. Since heterozygous mutation of *BRCA* genes increases the risk of breast cancer, precautionary attention should be taken for factors able to cause circadian disruption in *BRCA* mutation carriers [68]. Occupational Physicians should carefully evaluate the exposure to shift work, in particular to shift schedules that have frequent, long-term or a large number of night shifts over a lifetime, in *BRCA* mutation carriers.

## 4. Materials and Methods 

### 4.1. Participants and Sampling

#### 4.1.1. Healthy Volunteers

We enrolled 15 healthy subjects (eight males and seven females) aged 27–40 [mean ± standard deviation (SD): 33.1 ± 4.4 years]. Two subjects were smokers, all subjects drank a coffee every morning (range: 1–3 coffees/day) and did not drink alcohol daily. All subjects filled out a questionnaire that included their informed consent. The study was carried out according to the Declaration of Helsinki. The samples were processed under the approval (Prot. No. 737) of the Ethical Committee of Catania, Italy.

All subjects were required to have had regular sleep/wake patterns and no family history of breast, ovary, prostate, or pancreatic cancer. Their health status was assessed by a physical examination. None of the subjects had traveled across time zones or had been on medication in the past 2 months. Seven days prior to being admitted into the laboratory, subjects maintained their daily routines and slept for 8 h at regular times each night in the dark at home. Subjects entered the laboratory at 8:00 A.M. and remained there for a 24-hour period. Subjects were allowed to move, eat and drink ad libitum from 8:00 A.M. to 12:00 A.M. (awake time) and slept in the same room from 12:00 A.M. to 8:00 A.M. (sleep time). Environmental conditions were the same as a previous study [8]. Specifically, light intensity in the laboratory was measured at the eye level by Minolta Chroma Meter CL-100 (Minolta Camera Company, Ltd. of Osaka, Japan). While the volunteers were awake, the light intensity was 407.7 ± 112.5 lux (mean ± SD) that came from 4000K fluorescent lamps (Osram Lumilux, Osram, Munich, Germany). During sleep time, the light intensity was 2.6 ± 2.2 lux (mean ± SD) coming from a bulb emitting red (700K) light (Philips PAR38 IR, Philips Lighting, Eindhoven, The Netherlands). Room temperature was maintained at 22 ± 1 °C during the study. Blood was collected in an EDTA glass tube every 4 h for a 24-hour period (8:00 A.M., 12:00 P.M., 4:00 P.M., 8:00 P.M., 12:00 A.M., 4:00 A.M. and 8:00 A.M.). Immediately following blood sampling, lymphocytes and plasma were collected. Nocturnal samples (4:00 A.M.) were obtained under a light intensity of 49.1 ± 8.7 lux provided by a bulb emitting red (700 K) light (Philips PAR38 IR, Philips Lighting, Eindhoven, The Netherlands). The circadian synchronization of each subject was verified by assessing the rhythms of plasma melatonin and cortisol levels and clock genes expression (*BMAL1, PER2, PER3, REVERB*–α) in lymphocytes.

#### 4.1.2. Shift and Daytime Workers

*BRCA1* and *BRCA2* genes were further investigated in the lymphocytes of a sample of shift and daytime workers. We enrolled 50 shift workers and 50 daytime workers among healthcare workers of the Regional Hospital of Ancona, Italy. The shift workers schedule was as follows: 8:00 A.M.–2:00 P.M. alternating with 2:00 P.M.–8:00 P.M. 6 days per week and 5–6 nights (8:00 P.M.–8:00 A.M.) per month. The work schedule of daytime workers was from 8:00 A.M. to 2:00 P.M. 6 days per week. All workers were enrolled during the periodic medical examinations required by Italian Law and informed about the aims and modalities of the study, while obtaining their consent. As part of the standard occupational health surveillance, the study needed no formal approval by the local ethics committee. Nevertheless, the committee was consulted and it granted an informal authorization. Workers were selected based on the following criteria investigated during medical examination: female sex; no family history of breast, ovary, prostate or pancreatic cancer; no current treatment with drugs; and a negative history of psychiatric disorders, degenerative or cardiovascular diseases, insomnia, chronic viral infections, tumor or autoimmune diseases. Shift workers had to be assigned for at least 2 years to the current shift schedule including at least 50 night shifts per year without schedule breaks during the previous 2 months. Daytime workers must have had a routine sleep and wake schedule and no episode of sleep deprivation for at least 3 weeks prior to the study.

The participants were asked to fill in a questionnaire enquiring about their sleep habits and their light exposure. The chronotype was assessed by the “Morningness-Eveningness Questionnaire” (MEQ) [69], a 19-item questionnaire with a total score ranging from 16 to 86 extensively used in adults and workers [61,70,71,72,73]. Social jet lag has been operationalized as the absolute difference between midsleep on free days and midsleep on workdays [74]. Light exposure was estimated investigating three parameters: minutes usually exposed to sunlight, minutes of use of video display devices after dinner, and home light intensity (low/medium/high).

For both shift and daytime workers, fasting blood sampling was performed at 9:00 A.M. Since the study was part of the occupational health surveillance, the sampling time could not be changed. All workers were sampled after a day off. In that day, the workers were as comparable as possible, preventing acute alterations in shift workers due to the exposure of light at night and sleep deprivation associated with the night shift. Wake up time on the day of blood sampling was investigated. Sampling of all workers was completed in a short period (November 2018; local sunrise time: 6:40 A.M.–7:20 A.M., local sunset time: 5:00 P.M.–4:30 P.M.) to limit possible differences in time of sunlight exposure during the year. Some enrolled subjects were excluded from the study (*n* = 6 shift workers, *n* = 5 daytime workers) since they did not meet the selection criteria at the time of blood sampling. Samples were processed immediately after collection.

### 4.2. Melatonin and Cortisol Assay

Plasma levels of melatonin and cortisol were determined by enzyme immunoassay kits according to the manufacturer’s instructions (Human MT ELISA kit, eBioscience, San Diego, CA, USA and DetectX Cortisol ELISA kit, Arbor Assays, Ann Arbor, MI, USA, respectively). All samples were measured in duplicate. Samples from each subject were assayed in the same batch. The inter- and intra-assay variations of these analyses were all < 10%.

### 4.3. Gene Expression Analysis

Lymphocytes were isolated using a density gradient separation medium (Cedarlane Laboratories LTD., Hornby, ON, Canada) and stored at −80 °C until RNA extraction. Since peripheral blood mononuclear cells are a heterogeneous population of different cell types whose relative contribution may change between subjects and by time of day [75,76], we chose to investigate gene expression in only lymphocytes to obtain more specific data. The isolation of total RNA was performed using the RNeasy Mini Kit (QIAGEN, Hilden, Germany) according to the manufacturer’s instructions. RNA quality and quantification were evaluated with a Nanodrop 1000 spectrophotometer (Thermo Scientific, Wilmington, DE, USA). cDNA was synthesized according to the High-Capacity cDNA Reverse Transcription Kit protocol (Applied Biosystems, Foster City, CA, USA). The genes investigated were: *BMAL1*, *PER2*, *PER3*, *REV-ERBα* (clock genes) and *BRCA1* and *BRCA2* genes for the study of healthy volunteers, and *BRCA1, BRCA2* and *PER2* genes for the determinations on shift and daytime workers. Gene expression was analyzed in duplicate by real-time quantitative PCR using the FluoCycle II SYBR Master Mix (Euroclone S.p.A., Pero, Italy). Specific primer sets were obtained from IDT (Integrated DNA Technologies Inc., Coralville, IA, USA). Glyceraldehyde-3-phosphate dehydrogenase (*GAPDH*) was used as endogenous control. The relative mRNA expression levels were calculated applying the following equation: 2^−ΔCt^ [77].

### 4.4. Statistical Analysis

A total sample size of *n* = 10 volunteers was calculated a priori to detect significant differences with an effect size of 0.60, a power > 0.80, and a α = 0.05 (two-tailed) for all the variables studied. The theoretical sample size was increased by 50% in order to include a satisfactory final number of participants. Variables were expressed as mean ± SD. Data about gene expression in healthy volunteers were showed as percentages of relative mRNA expression where the highest mean value (acrophase) of each gene is set to 100%. ANOVA repeated measures was performed to analyze repeated measures at different time points with LSD as post-hoc test. Mauchly’s test was performed to verify the sphericity assumption. Cosinor analysis was applied to study circadian rhythmicity. Student’s *t*-Test was used to test differences of independent measures between two groups. The Chi-square test was used to test dichotomous parameters. Pearson correlation test was applied to analyze relationships between continuous parameters. Linear regression analysis was used to assess *BRCA1* and *BRCA2* expression as well as cortisol level and *PER2* expression in the shift and daytime workers. Explanatory variables associated with the outcome, at a significance of ≤0.20 at univariate analysis, were included as independent variables in the multivariate analysis. Wake up time on the day of blood sampling was considered as a potential confounder a priori in the multivariate analysis. Statistical significance was set at *p* < 0.05, and statistical tests were two-sided. We analyzed our data by Statistical Package Social Sciences (version 19) software (SPSS, Chicago, IL, USA) and Circadian software [78].

## 5. Conclusions

Both *BRCA1* and *BRCA2* gene expression were characterized by diurnal variability with the peak at midday and the minimum at midnight. This finding suggests a relation of DSB repair system with biological clock.

Lower levels of *BRCA1* and *BRCA2* expression were found in the morning in a group of shift workers. It is unknown if this evidence is caused by a reduced amplitude and/or a phase misalignment, but in any case, it may be one of the potential factors related to the higher risk of breast cancer.

Since this study cannot rule out possible compensatory mechanisms and RNA expression in lymphocytes may not represent the breast tissue, further studies on the effect of shift work on DSB repair pathway are encouraged. 

## Figures and Tables

**Table 1:** Demographics and habits characteristics of shift and daytime workers.

Parameters	Shift Workers (*n* = 44)		%	Daytime Workers (*n* = 45)		%	*p*-Value
	Mean	SD		Mean	SD		
Age (years)	40.2	9.5		43.0	10.7		0.196
Job Seniority (years)	15.9	5.4		17.1	4.7		0.266
Shift Work Seniority (years)	15.9	5.4					
Night Shift Work (nights per month)	5.6	1.3					
Smokers (%)			20.5			17.8	0.748
BMI	23.6	3.4		23.5	7.5		0.936
Physical Activity (hours/week)	3.1	3.2		2.7	2.0		0.480
Chronotype (MEQ score)	53.7	7.1		58.3	7.9		0.005
Wake-up Time on Blood Sampling Day	6:22	0:20		6:18	0:42		0.569
Social Jet Lag (minutes)	52.4	31.1		36.7	20.7		0.006
Exposure to Sunlight (minutes)	100.9	69.7		105.3	71.4		0.769
Use of Video Display Devices after Dinner (minutes)	104.1	59.7		99.0	60.1		0.689
Home Light Intensity (low/medium/high)			15.9/79.5/4.6			22.2/51.1/26.7	0.006

**Table 2:** Pearson correlation among *BRCA1* and *BRCA2* gene expression, cortisol plasma levels, *PER2* expression, and nights per month of night-shift work. * = *p* < 0.05.

Parameters	*BRCA1*	*BRCA2*	Cortisol	*PER2*	Night Shift Work (nights per month)
*BRCA1*	1	0.830 *	−0.005	0.496 *	−0.284 *
*BRCA2*	0.830 *	1	0.107	0.477 *	−0.309 *
Cortisol	−0.005	0.107	1	0.067	−0.127
*PER2*	0.496 *	0.477 *	0.067	1	−0.267 *
Night-shift work (nights per month)	−0.284 *	−0.309 *	−0.127	−0.267 *	1

**Table 3:** Effect of shift work on *BRCA1* and *BRCA2* gene expression, on cortisol and on *PER2* expression, unadjusted and adjusted for covariates. Results of linear regression analysis. Bold: highlight significant variables.

Parameters	*BRCA1* Gene Expression				*BRCA2* Gene Expression			Cortisol					*PER2* Gene Expression			
	β	*p*-Value	β	*p*-Value	β	*p*-Value	β	*p*-Value	β	*p*-Value	β	*p*-Value	β	*p*-Value	β	*p*-Value
Shift Work	**−0.258**	**0.016**	**−0.298**	**0.012**	**−0.282**	**0.008**	**−0.296**	**0.012**	−0.115	0.310	−0.177	0.170	**−0.269**	**0.011**	**−0.262**	**0.031**
Age	—	—	−0.057	0.633	—	—	−0.093	0.492	—	—	−0.179	0.173	—	—	−0.004	0.976
Chronotype	—	—	−0.231	0.072	—	—	−0.085	0.500	—	—	−0.196	0.151	—	—	−0.118	0.356
Wake Up Time on Blood Sampling Day	—	—	**−0.257**	**0.039**	—	—	**−0.270**	**0.028**	—	—	−0.139	0.288	—	—	−0.133	0.297
Social Jet Lag	—	—	0.035	0.775	—	—	0.083	0.487	—	—	−0.205	0.120	—	—	0.011	0.927
Home Light Intensity	—	—	0.092	0.428	—	—	0.131	0.253	—	—	−0.061	0.622	—	—	0.034	0.778
